# M2 macrophage inhibits the antitumor effects of Lenvatinib on intrahepatic cholangiocarcinoma

**DOI:** 10.3389/fimmu.2023.1251648

**Published:** 2023-09-22

**Authors:** Long Yang, Pinsheng Han, Tao Cui, Yu Miao, Tianyu Zhao, Zilin Cui, Yijia Chen, Hao Chi, Jieying Zhang, Yamin Zhang

**Affiliations:** ^1^ Department of Hepatobiliary Surgery, Tianjin First Central Hospital, School of Medicine, Nankai University, Tianjin, China; ^2^ School of Medicine, Nankai University, Tianjin, China; ^3^ State Key Laboratory of Drug Delivery Technology and Pharmacokinetics, Tianjin Institute of Pharmaceutical Research, Tianjin, China; ^4^ Research Unit for Drug Metabolism, Chinese Academy of Medical Sciences, Beijing, China; ^5^ Clinical Medical College, Southwest Medical University, Luzhou, China; ^6^ Department of Tuina, First Teaching Hospital of Tianjin University of Traditional Chinese Medicine, Tianjin, China; ^7^ Department of Tuina, National Clinical Research Center for Chinese Medicine Acupuncture and Moxibustion, Tianjin, China

**Keywords:** tumor microenvironment, immune regulatory, tumor-associated macrophages, cholangiocarcinoma, lenvatinib

## Abstract

**Background and objectives:**

The relationship between the tumor microenvironment and the network of key signaling pathways in cancer plays a key role in the occurrence and development of tumors. Tumor-associated macrophages (TAMs) are important inflammatory cells in the tumor microenvironment and play an important role in tumorigenesis and progression. Macrophages in malignant tumors, mainly the M2 subtype, promote tumor progression by producing cytokines and down-regulating anti-inflammatory immune responses. Several articles have investigated the effect of macrophages on the sensitivity of cancer chemotherapeutic agents, but few such articles have been reported in cholangiocarcinoma, so we investigated the effect of M2 macrophage on the sensitivity of cholangiocarcinoma cells to Lenvatinib compared to M1.

**Methods:**

THP-1 monocytes were polarized to M0 macrophage by phorbol 12-myristate 13-acetate (PMA) and then induced to differentiate into M1 and M2 macrophages by LPS, IFN-γ and IL-4 and IL-13, respectively. Macrophages and cholangiocarcinoma cells were co-cultured prior to 24 hours of Lenvatinib administration, cancer cell apoptosis was detected by western-blot, FACS analysis of Annexin V and PI staining. Furthermore, we use xCELLigence RTCA SP Instrument (ACEA Bio-sciences) to monitor cell viability of Lenvatinib administration in co-culture of cholangiocarcinoma cells and macrophages. After tumorigenesis in immunodeficient mice, Lenvatinib was administered, and the effects of M2 on biological characteristics of cholangiocarcinoma cells were investigated by immuno-histochemistry.

**Results:**

mRNA and protein expression of M1 and M2 markers confirmed the polarization of THP-1 derived macrophages, which provided a successful and efficient model of monocyte polarization to TAMs. Lenvatinib-induced apoptosis of cholangiocarcinoma cells was significantly reduced when co-cultured with M2 macrophage, whereas apoptosis of cholangiocarcinoma cells co-cultured with M1 macrophage was increased. In the CDX model, Lenvatinib-induced cancer cell apoptosis was markedly reduced, and proliferative cells increased in the presence of M2 macrophages. Angiogenesis related factors was significantly increased in cholangiocarcinoma cells co-cultured with M2.

**Conclusion:**

Compared with M1, M2 macrophages can inhibit the anti-tumor effect of Lenvatinib on cholangiocarcinoma through immune regulation, which may be related to the tumor angiogenesis factor effect of M2 macrophage.

## 1 Introduction

Intrahepatic cholangiocarcinoma (ICC) is originally a relatively rare primary liver cancer originating from intrahepatic bile duct epithelial cells. In the past 10 to 20 years, ICC has become a growing concern due to its increasing incidence and mortality worldwide (1). The 1-year and 5-year overall survival rates of ICC patients have been estimated to be approximately 30% and 18%, respectively (2). Several studies have shown that ICC is more likely to metastasize through the lymphatic route and is more aggressive than Hepatocellular carcinoma (HCC) (3). Therefore, although radical resection can be performed at the early stage of ICC, the high postoperative recurrence rate makes the treatment of ICC a great challenge. In recent years, with the emergence of new targeted drugs and immune checkpoint inhibitors, patients with recurrent and unresectable ICC can get more effective treatment.

As a multi-target tyrosine kinase inhibitor, Lenvatinib can control the growth and angiogenesis of malignant tumors by inhibiting many receptors such as vascular endothelial growth factor receptor (VEGFR), fibroblast growth factor receptor (FGFR) and platelet-derived growth factor receptor (PDGFR) (4). At present, Lenvatinib is approved for the first-line treatment of advanced HCC (5). Some scholars believe that because ICC and HCC live in similar environments (both in the liver) and originate from the same organ, they are highly homologous, and they respond similarly to antitumor drugs, it can be predicted that Lenvatinib is equally effective against ICC. Xia Yan et al. confirmed that Lenvatinib has a good anti-tumor effect on ICC by cell experiment and PDX model (6). Recent clinical studies have also shown that patients can benefit from the treatment of cholangiocarcinoma with Lenvatinib. At the same time, with the advent of immune checkpoint inhibitors, the combination of Lenvatinib and immunotherapy has become a new research hotspot for the treatment of ICC (7–9). Although some studies have confirmed the effectiveness of Lenvatinib in some ICC tumors, the efficiency of its monotherapy remains low and the reasons for resistance are unclear.

TAM, one of the most important immune cells in tumor microenvironment (TME), plays an important role in tumor proliferation, invasion, metastasis, and angiogenesis (10). The macrophages recruited to tumor tissue are usually divided into two types, one is M1 macrophage, which activates immune response, phagocytizes and kills cancer cells, and acts as inhibitor of cancer cell activity. The other kind of M2 macrophage can promote tissue repair and tumor angiogenesis, which plays a role in promoting tumor activity. M1 and M2 macrophages continue to transform under the influence of tumor microenvironment, which is called “macrophage polarization”. Mantovani et al. described these two macrophage phenotypes as two extremes of different functions in the process of transformation (11).

Lipopolysaccharide (LPS) and Interferon-γ (IFN-γ) induce M0 macrophage into M1 macrophage *in vitro*, while IL-4 and IL-13 induce M0 macrophage into M2 macrophage (12). M1 macrophage can recognize tumor cells differently from normal tissues by cell surface antigens and produce tumor cell killing factors such as nitric oxide and reactive oxygen species. M2 macrophage can secrete cytokines such as IL-10, TGF- β, PGE2, VEGF and MMPs, which play a key role in tumor angiogenesis and metastasis (13). In most tumors, TAM is generally considered to be closer to M2 macrophage (14).

The effect of M1 and M2 macrophages on the treatment of ICC is not clear, so in order to study the effect of two phenotypic macrophages on the sensitivity of lenvatinib to the treatment of intrahepatic cholangiocarcinoma, we co-cultured two kinds of macrophages with intrahepatic cholangiocarcinoma cells (RBE) and used severe immunodeficient mice (NSG) to establish a tumor-bearing model combined with RBE and macrophages to observe TAM. In particular, the effects of M2 macrophage on the proliferation, apoptosis and angiogenesis of establishing a TAM-PDX model using NSG mice in order to provide new therapeutic targets and clinical strategies for the treatment of ICC.

## 2 Materials and methods

### 2.1 Cell culture

In this study, the human monocytic cell line THP-1(National Experimental Cell Resource Sharing Platform, 1101HUM-PUMC000057) and the human intrahepatic cholangiocarcinoma cell line RBE (National Experimental Cell Resource Sharing Platform, 1101HUM-PUMC000675)were selected, and the medium used was RPMI-1640 medium (Gibco) (containing 10% fetal bovine serum(FBS, Sigma)).

### 2.2 Macrophage polarization

When 150ng/ml phorbol ester(PMA)(Sigma) was added to THP-1 cells for 48 hours, most of the cells adhered to the wall, the size of the cells increased and antennae grew, which was considered to polarize to M0 macrophage. On the basis of M0 macrophage, 1 μg/ml LPS(R&D Systems) and 10 ng/ml IFN-γ(Sigma)were used to induce them to polarize into M1 macrophage. After 48 hours of incubation, the supernatants were collected and the levels of IL-6 and IL-1β were detected by ELISA kit(R&D Systems). On the basis of M0 macrophage, 20ng/ml IL-4(R&D Systems) and 5ng/ml IL-13(R&D Systems)were used to induce them to polarize into M2 macrophage.

### 2.3 ELISA

The M1 macrophage solution was diluted 100 times. The concentrated wash solution was then diluted with double distilled water and the assay kit should be equilibrated at room temperature prior to the experiment. 5ul standard and 5ul diluted fetal bovine serum were added sequentially to the pore of the reaction plate and mixed for 10 seconds. We added 200ul Biotin antihuman IL-6 or IL-1 β to each well and mixed for 30s. Incubated at 37°C for 30 minutes. After cleaning the reaction plate with detergent, we added 200ulHRP to each well, mixed gently for 10sm and incubated at 37°C for 30 minutes. After repeatedly cleaning the reaction plate 5 times, 100ul TMB chromogenic solution was added to each well, mixed for 10s and then placed in a dark place to react for 20 minutes. The terminating solution of 100ul was added to each well and mixed for 30s before OD value was read at the 450nm wavelength within 15 minutes. According to the OD value, the corresponding concentration can be found on the standard curve.

### 2.4 M2 macrophage immunofluorescence staining

M2 macrophage were fixed with 4% paraformaldehyde solution at 4°C for 30 minutes and washed with PBS solution. After dripping goat serum on the climbing tablet, it was sealed at room temperature for 1 hour, and then washed again with PBS solution. It was incubated with CD163(Abcam)and CD206(Abcam)overnight at 4°C with the primary antibody 1:100 diluted. Cells were washed three times with PBS and then incubated for 2h with the secondary antibody(Abcam)at 1:1000 dilution at room temperature. Cells were then washed three times with PBS, and DAPI was added and incubated for 5 minutes protected from light. Finally, the images were observed and collected under fluorescence microscope.

### 2.5 RT-qPCR

The RNA of M0, M1 and M2 macrophages obtained by TRIzol method was isolated with trichloromethane, precipitated with isopropanol, washed with 75% ethanol and dissolved in DEPC water to obtain purified RNA. RNA was reverse transcribed into cDNA by reverse transcription reagent (Roche). Amplification reaction assays contained SYBRGreen PCR Master Mix (Applied Biosystem) and primers (IDT).The reaction conditions were set as follows: 95°C, pre-denaturation for 10 minutes, denaturation for 15s, annealing extension for 1min at 58°C, 40 cycles, and data collection was performed at the end of annealing extension. After the amplification, the specificity of the product was analyzed by melting curve reaction. The results were analyzed by 2-CT method, and GAPDH was used as an internal reference.

### 2.6 Transwell cell co -culture

**Figure 1:**
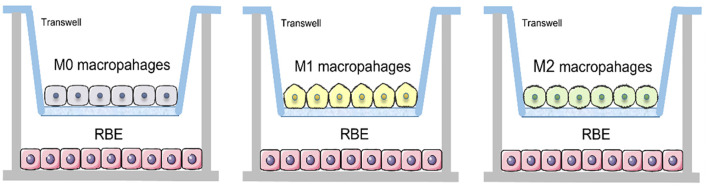
Schematic of Transwell co-culture of RBE cells with three different subtypes of macrophages.

THP-1 cells were induced into M0, M1 and M2 macrophages in Transwell nested membranes (membrane pore size 0.4 μm) (corning) according to the above steps. Then removed the upper layer of membranes and place them on the other Transwell nested membranes where 1.5ml of 3×10^5^ RBE cells were placed in the lower layer and co-cultured for 48 hours (**Figure 1**).

### 2.7 RTCA screening for effective drug concentrations

50 μl of medium was added to each well of the 96-well U base plate with low adsorption, and the baseline was assayed by xCELLigence RTCA eSight Instrument. 100 μl of RBE cells at a density of 5×10^4^ cells/ml were added to each well of a 96-well U-backed plate with low adsorption. The wells were placed in a biosafety cabinet at room temperature for 30 minutes and then moved into a real-time label-free dynamic cell analyzer. The cell index was recorded, the cell proliferation curve was drawn, and pictures were taken every hour. After a 1.5-fold expansion of the cells, 100 μl/well of different concentrations of Lenvatinib (0nM, 100nM, 0.3uM, 1uM, 3uM, 10uM, 30uM, 90uM) were added, and eight compound wells were used. The cell proliferation curve was plotted by the instrument software, and pictures were taken every hour to observe the effects of different concentrations of drugs on the growth and proliferation of RBE cells.

### 2.8 Experimental group

Through the above-mentioned experiments, we selected the most suitable concentration of Lenvatinib for RBE cells, and then established five groups, namely, RBE group, RBE and M0 co-culture group, RBE and M1 co-culture group, RBE and M2 co-culture group, respectively, and set up a simple RBE group without drug as control.

### 2.9 Western blot

After 48 hours of incubation, it was washed with PBS solution, and then added to the cleavage buffer containing protein phosphatase inhibitor complex and protease inhibitor. The pyrolysis products were clarified by centrifugation and quantified by Bradford method. The pyrolysis product was then boiled for 5min and sampled onto a 10% polyacrylamide gel and transferred to a PVDF membrane. Antibodies to Caspase3, Bax, Bcl2, Bcl-xl and Tubulin(1:1000) (all purchased from Abcam)were blocked with 5% skim milk and incubated overnight at 4°C. After washing with TBS-T, the Strips were incubated at room temperature with anti-rabbit polyclonal antibody (1:5000) or anti-mouse antibody (1:5000) polyclonal antibodies for 2 hours. Immobilon Western chemiluminescence solution was added, and the fluorescence intensity was detected by ultra-sensitive chemiluminescence imager and performing quantitative testing using Image J.

### 2.10 Analysis of apoptosis in each group by flow cytometry

Each group of cells was digested with EDTA-free trypsin, centrifuged and washed twice with PBS solution, then resuscitated with 500 μl Binding buffer, then mixed sequentially with 5 μl Annexin V-EGFP and 5 μl Pi stain, incubated for 10min at room temperature, and then immediately detected by flow cytometry. Annexin V-FITC Cell Apoptosis Detection Kit was purchased from Signalway Antibody.

### 2.11 RTCA observation of the effect of Lenvatinib on cell proliferation in each group

The 1640 culture medium of the three co-culture groups was replaced with the corresponding macrophage culture medium and placed into the low adsorption 96-well U base plate, respectively. The backplane was placed in a real-time dynamic cell analyzer according to the RTCA operation steps mentioned above. To start step 3, cell indices were recorded and photographed hourly to observe the effect of drugs on the proliferation of tumor cells in each group. Record the number of cells in each visual field after 36 hours, and draw a column diagram after repeating the experiment three times.

### 2.12 Establishment of CDX model with NSG mice

In this study, male NOD-SSCID-IL2rg-/- (NSG) mice from 4 to 6 weeks of age were selected to establish an RBE+M2 macrophage xenograft mode. The animal experiment was divided into RBE group, RBE and M2 co-culture group, RBE+ Lenvatinib group, RBE and M2 co-culture + Lenvatinib group, with 5 mice in each group. All the mice were raised in the SPF animal room of the Biotechnology Center of Tianjin Pharmaceutical Research Institute. The temperature was about (25 ± 3)°C, the humidity was 40% or 60%, light was maintained for 12 hours per day, free feeding, dressing was changed regularly, and the animal room was kept clean. In RBE group, 0.1ml of 3×10^7^ cells/ml RBE cell suspension was inoculated into the left and right axilla of mice, and in RBE and M2 co-culture group, 0.1ml of 3×10^7^ cells/ml RBE cell suspension and 0.1ml of 1.5×10^7^ cells/ml M2 macrophage suspension were mixed and inoculated into the axilla of mice. The mice in the two groups began to develop subcutaneous tumors at about 1 week, then intragastric administration of Lenvatinib to Lenvatinib group mice, and the mice were killed by cervical dislocation 3 weeks later. The longest diameter (A) and shortest diameter (B) of the transplanted tumor were measured by Peira TM900. According to the formula, the tumor volume was calculated as (A×B^2^)/2(mm^3^).

### 2.13 Drug sensitivity test *in vivo*

The tumor formation began about 1 week after subcutaneous inoculation of the cell suspension, and 1.4 mg/kg Lenvatinib was given daily to the stomach for 3 weeks. Mice were executed after 4 weeks, and the transplanted tumor tissue was excised for examination. There was no mice death in the process of tumor formation and administration.

### 2.14 Immunohistochemical staining of transplanted tumor tissues

The fresh transplanted tumor tissue was fixed in a 10% formalin fixed solution for 24 hours, dehydrated in gradient alcohol, and then the tissue blocks were placed transparently in xylene. The transparent tissue blocks were embedded in liquid paraffin, sliced after solidification, and baked on glass slides. Prior to staining, dehydration was performed with xylene and gradient alcohol dewaxing, and distilled water washing followed by inactivation of endogenous peroxidase with 3% hydrogen peroxide solution and antigen repair with sodium citrate buffer. After serum sealing, first antibody (caspase3, caspase8, Bcl-2, VEGF, VEGFR-1, PCNA, Ki-67, CD163, CD206, Fas, Survivin) (1:200)(all purchased from Abcam)was added to incubate overnight at 4°C and rinsed 3 times with PBS. Then we added second antibody: anti-rabbit polyclonal antibody (1:5000) or anti-mouse antibody (1:5000) polyclonal antibodies, incubated at 37°C for 30 minutes and rinsed 3 times. We dropped the chromogenic solution, hematoxylin dye re-dyeing, dehydration, transparent with xylene, and gum sealed microscopic examination.

### 2.15 Statistical analysis

T-test was used for unpaired continuous variable data w that conformed to a normal distribution. One-way Analysis of Variance (ANOVA) was used for data comparison between multiple groups (more than two groups). All statistical analyses were carried out using SPSS 23.0 software. The statistical analysis data were considered to be statistically significant (P<0.05).

## 3 Results

### 3.1 Polarization of M0 macrophage detected by qRT-PCR

**Figure 2:**
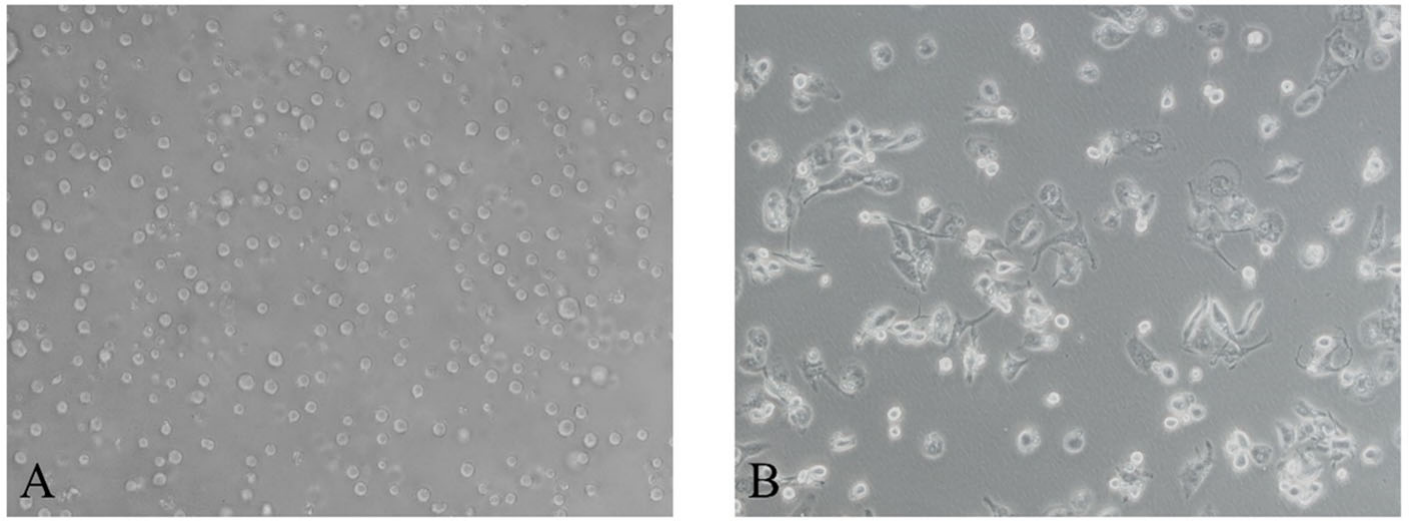
After induction with PMA for 48 hours, Morphology of human monocytes THP-1 is polarized to M0 macrophage. **(A)** THP-1 monocytes (200×). **(B)** M0 macrophage (200×).

**Figure 3:**
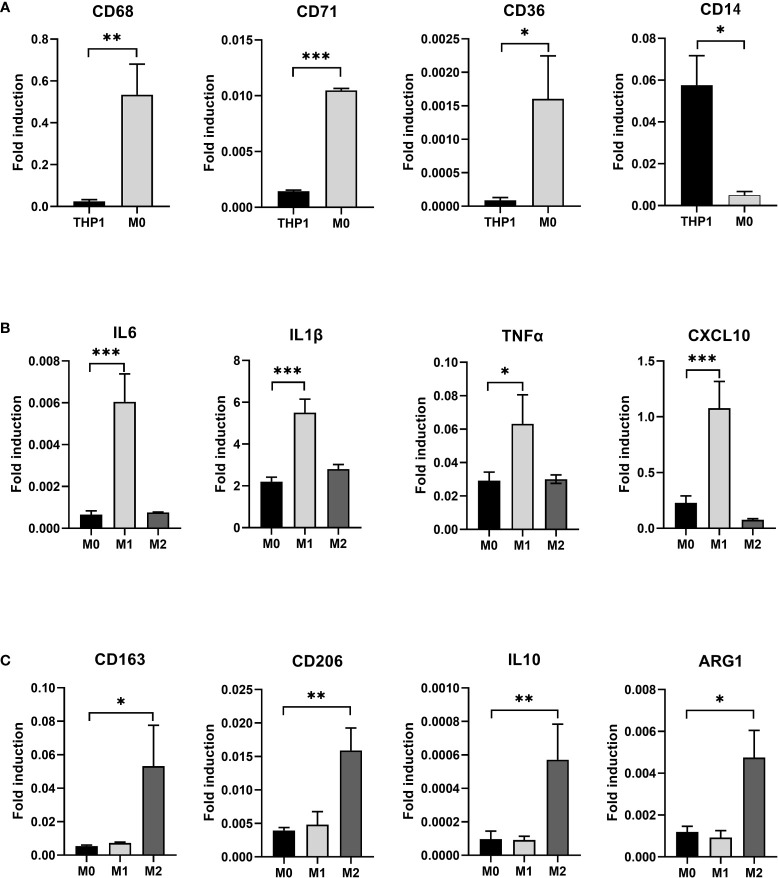
**(A)** After induction with PMA for 48 hours, THP-1 is polarized to M0 macrophage. RT-PCR detected the expression of THP-1 and induced M0 macrophage CD68, CD71, CD36, and CD14. **(B)** After induction with LPS and IFN-γ for 24 hours, M0 macrophage is polarized to M1 macrophage and after induction with IL-4 and IL-13 for 24 hours, M0 macrophage is polarized to M2 macrophage. RT-PCR detected the expression of M1 specific markers IL6, IL1β, TNFα, and CXCL10 in three types of macrophages. **(C)** RT-PCR detected the expression of M2 specific markers CD163, CD206, IL10, and ARG1 in three types of macrophages(* P<0.05, ** P<0.01, *** P<0.001).

After induction of THP-1 cells with PMA for 48 hours, the volume of cells gradually increased, and the morphology changed from suspension-like round cells to adherent and irregular polygonal cells (**Figure 2**). qRT-PCR after RNA reverse transcription showed a significant increase in the expression of CD68, CD71 and CD36 in M0, while the monocyte marker CD14 was significantly decreased. The difference was statistically significant, indicating that THP-1 monocytes successfully differentiated into M0 (**Figure 3A**).

### 3.2 M0 polarization into M1 and M2 macrophages

We exposed M0 macrophage to the medium containing 1μg/ml LPS and 10ng/ml IFN- γ for 24 hours to induce their differentiation into M1 macrophage, while the other group was exposed to the medium containing 20ng/ml IL-4 and 5ng/ml IL-13 to induce them to differentiate into M2 macrophage. Three types of macrophages were collected. qRT-PCR after RNA reverse transcription showed a significant increase in specific markers such as IL6, IL1β, TNFα and CXCL10 in M1 macrophage in the LPS+IFN-γ group (P<0.05) (**Figure 3B**). However, the special markers CD163, CD206, IL10 and ARG1 in IL-4+IL-13 group were significantly higher than those in control group (P<0.05) (**Figure 3C**).

**Figure 4:**
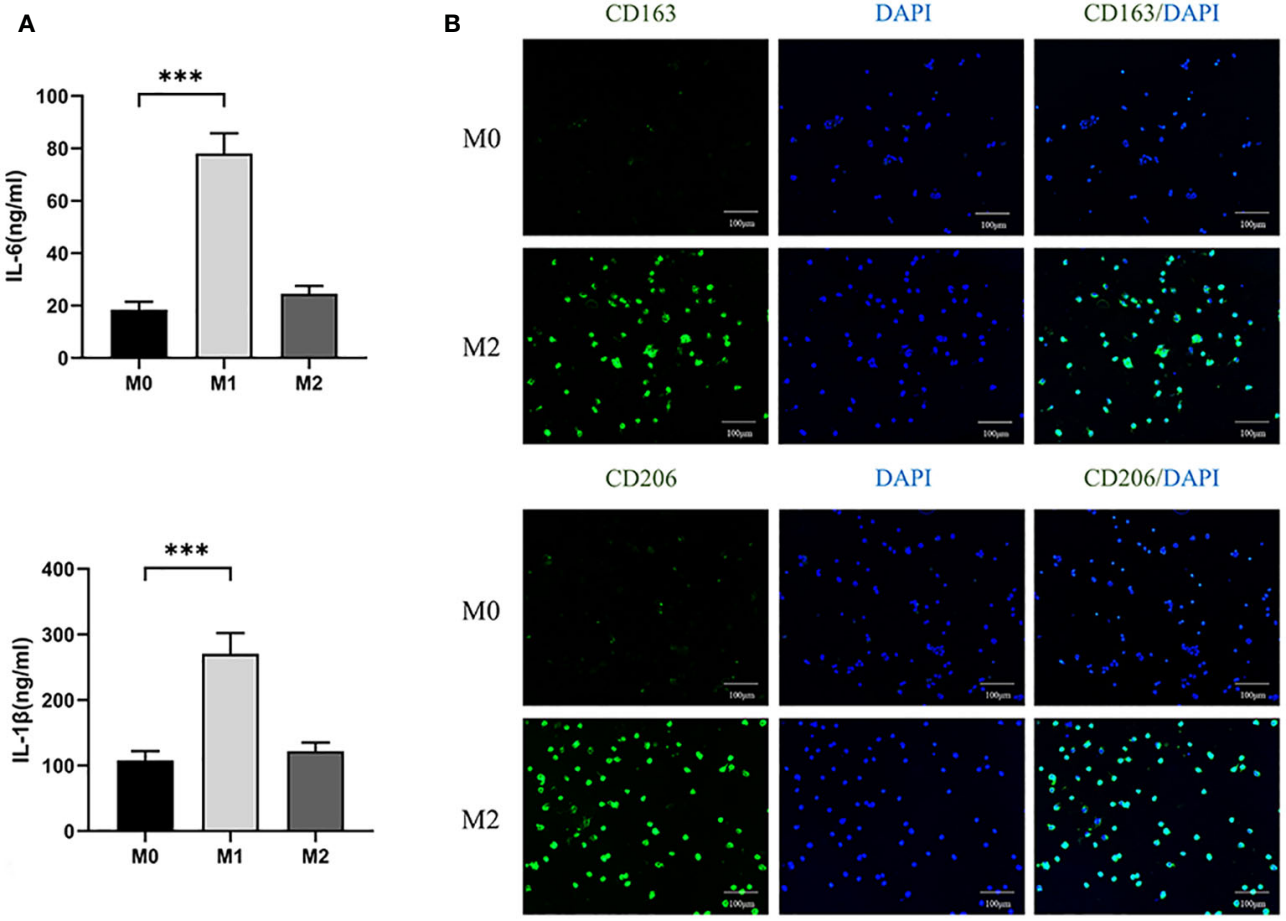
**(A)** ELISA analyzed the expression levels of IL-6 and IL-1β in three macrophages. (100×) (*** P<0.001). **(B)** Immunofluorescence analysis of expression of CD163 and CD206 in M0 and M2 macrophages.

In addition, we collected supernatants of three macrophage cultures and analyzed the levels of IL-6 and IL-1β secreted by M1 by ELISA. It was found that the expression of IL-6 and IL-1 β in M1 was higher than that in M0 (P<0.05) (**Figure 4A**). The results indicate that we successfully polarized M0 into M1.

After differentiating M0 macrophage into M2 macrophage, we used immunofluorescence to compare the expression of CD163 and CD206, specific markers of M2, between the two kinds of cells. The expression levels of M2 were found to be significantly higher than those of M0 (**Figure 4B**). The results showed that M0 were successfully polarized into M2.

### 3.3 Screening of lenvatinib concentration by RTCA eSight

**Figure 5:**
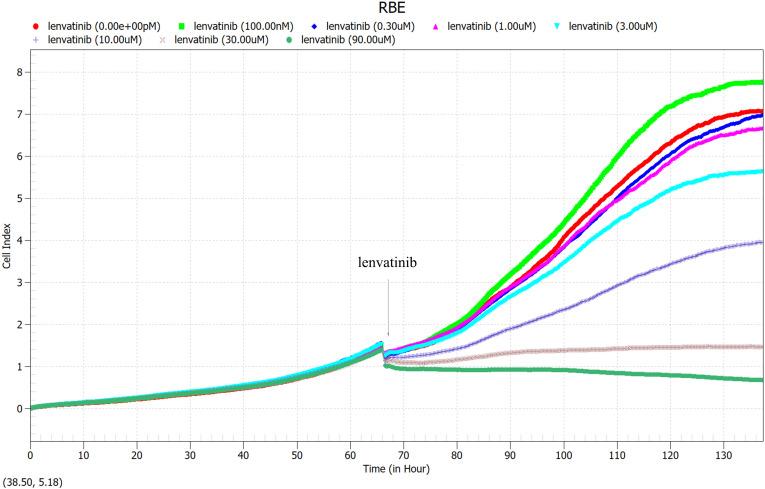
100 μl of RBE cells at a density of 5×10^4^ cells/ml were added to each well of a 96-well U-backed plate with low adsorption. RBE cells were cultured for 66 hours, then different concentrations of Lenvatinib (0nM, 100nM, 0.3uM, 1μM, 3μM, 10μM, 30μM, 90μM) were added to observe the proliferation curve of RBE cells. (Arrow: administration time).

**Figure 6:**
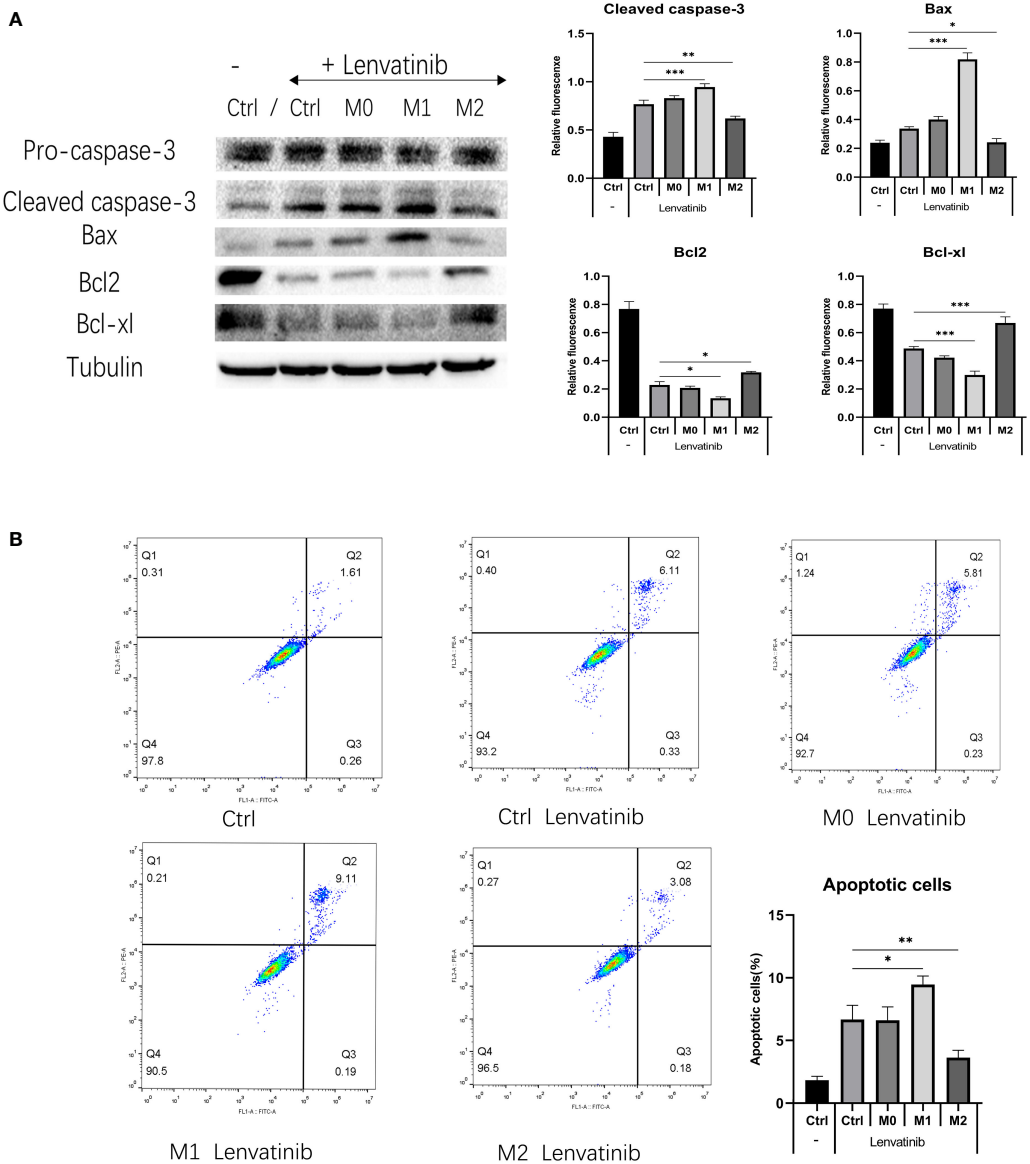
RBE cells were co-cultured with three kinds of polarized macrophages in a 0.4 μm Transwell chamber after administration of Lenvatinib for 48 hours. (Ctrl: control group) **(A)** Western Blot detects the expression of apoptotic-related proteins (caspase-3 and Bax) and anti-apoptosis related proteins (Bcl-2 and Bcl-xl) in each group of cells(*P<0.05, ** P<0.01, *** P<0.001). **(B)** Flow cytometry detects apoptosis of RBE cells in each group and plotted the histogram by replicating the experiment three times.(* P<0.05,** P<0.01).

RBE cells were cultured 66 hours in 96-well U-backed plate with low adsorption, different concentrations of Lenvatinib (0nM, 100nM, 0.3uM, 1μM, 3μM, 10μM, 30μM, 90μM) were added to observe the proliferation curve of RBE cells. Immediately after drug administration, the cell proliferation curve decreased, and RBE cells with different drug concentrations exhibited different proliferation rates as time progressed (**Figure 5**). As shown in **Figure 6**, the cell proliferation rate was the fastest in the 100nM group, while in the 30 μM group and 90 μM group, almost all of the cells died and did not proliferate due to the high drug concentration. Finally, according to the proliferation curve, we selected 10umol/L with a cell inhibition rate of about 40% as the concentration for the subsequent drug sensitivity test of Lenvatinib.

### 3.4 Apoptosis and expression of apoptosis-related proteins

RBE cells were co-cultured with three kinds of polarized macrophages in a 0.4 μm Transwell chamber. After 48 hours of treatment, the expression of apoptosis-related proteins caspase-3 and Bax in M1+RBE cells was significantly increased, whereas it was significantly decreased in M2+ RBE cells. The expression of anti-apoptosis related proteins Bcl-2 and Bcl-xl was significantly decreased in RBE cells treated with M1 and increased in RBE cells treated with M2 (**Figure 6A**). The results showed that M1 might promote the apoptosis of RBE cells induced by Lenvatinib, while M2 could inhibit its apoptotic effect. In addition, we detected the apoptosis of RBE cells in five groups by flow cytometry and plotted the histogram by replicating the experiment three times. (**Figure 6B**). Apoptosis was most significant in the M1+RBE group of RBE cells, while the number of apoptosis in the M2+RBE group was significantly lower than that in the control group, which was consistent with the results of western blot detection.

### 3.5 RTCA eSight observation of cell viability in each group

**Figure 7:**
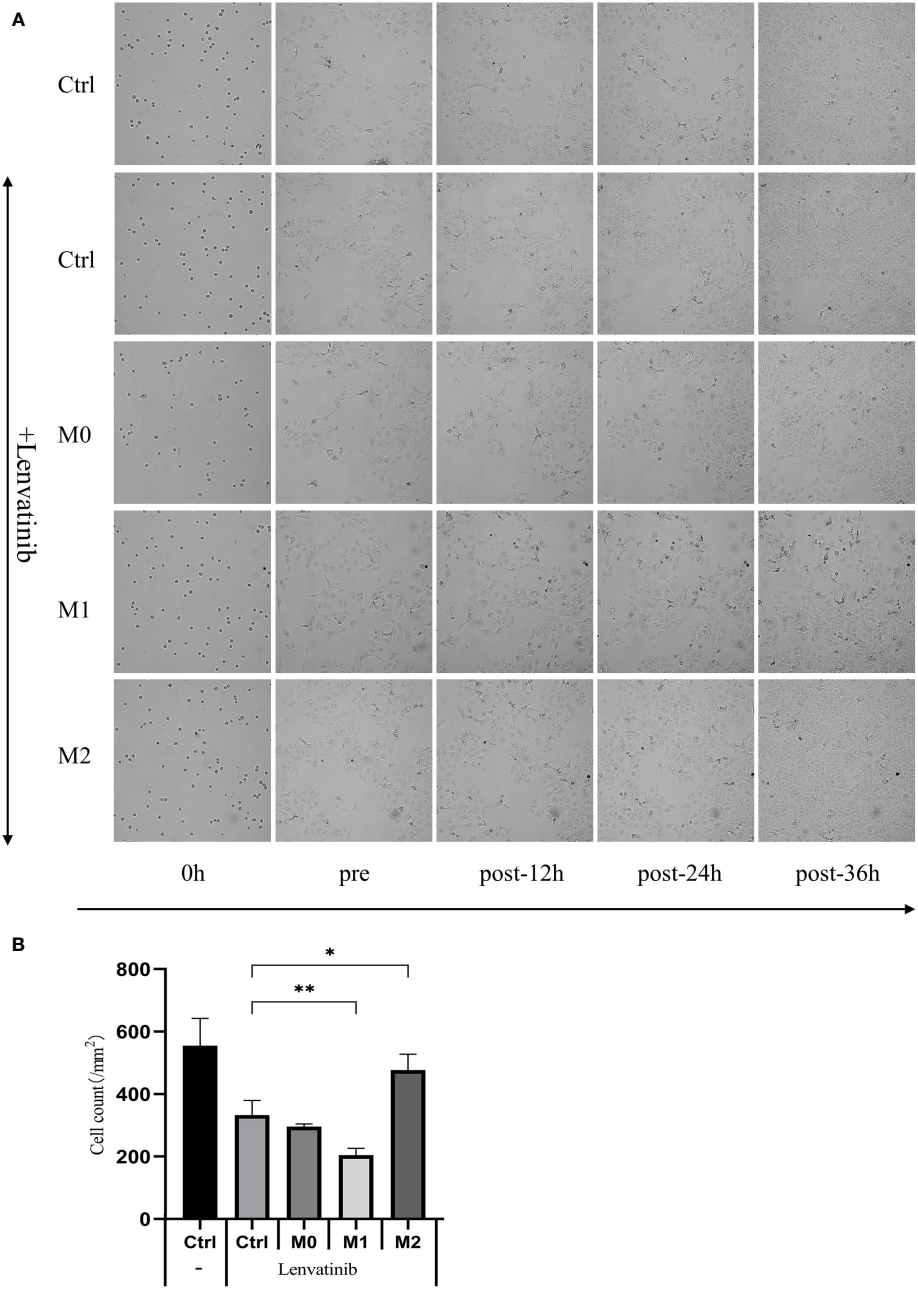
The supernatant of three kinds of macrophages was added to the low adsorption 96-well U floor with RBE cells and put into an RTCA eSight incubator. 3 μmol/L Lenvatinib was added to each group and pictures were taken every hour and observed continuously for 36 hours. (Ctrl: control group) **(A)** RTCA eSight dynamically observed the proliferation status of 5 groups of RBE cells in real time. **(B)** Counting the cells within each set of fields of view and plotted the histogram by replicating the experiment three times. (* P<0.05,** P<0.01).

The culture media of three kinds of macrophages were centrifuged, and the supernatant was added to the low adsorption 96-well U floor with RBE cells and put into an RTCA eSight incubator. 3μmol/L Lenvatinib was added to each group. Pictures were taken every hour and observed continuously for 36 hours. The results of RTCA eSight showed that compared with the control group, the proliferation of RBE cells was significantly inhibited in Lenvatinib. The proliferation rate of RBE cells co-cultured with M1 macrophage was significantly reduced, while the proliferation rate of RBE cells treated with M2 was significantly increased (**Figure 7A**) and counting the cells within each set of fields of view (**Figure 7B**). This suggests that M1 can further inhibit the proliferation of RBE cells, while M2 can promote the proliferation of RBE cells.

### 3.6 Immune regulatory effect of M2 macrophage on the growth of RBE transplanted tumor in CDX model

**Figure 8:**
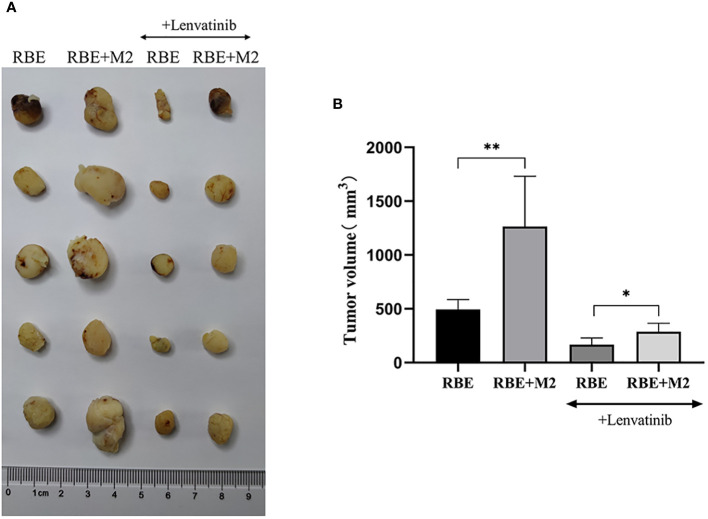
In RBE group, Subcutaneous injection 0.1ml of 3×10^7^ cells/ml RBE cell suspension, and in RBE and M2 co-culture group, Subcutaneous injection 0.1ml of 3×10^7^cells/ml RBE cell suspension and 0.1ml of 1.5×10^7^cells/ml M2 macrophage suspension. The mice in the four groups began to develop subcutaneous tumors at about 1 week, then intragastric administration of Lenvatinib to Lenvatinib group mice for 3 weeks, and the mice were killed by cervical dislocation and removed the transplanted tumor. **(A)** Picture for volume comparison of graft tumors in various groups of CDX models (n=5 per group). **(B)** Measuring tumor volume and plotted the histogram by replicating the experiment three times. (* P<0.05,** P<0.01).

By measuring the volume of transplanted tumors in NSG mice, it was found that among the four groups of transplanted tumors the RBE+M2 combined transplantation group (untreated) had the largest transplanted tumor volume and the RBE group had the smallest. Comparing the transplanted tumor volume, it was found that the transplanted tumor volume of RBE+M2 co-transplantation group was significantly larger than that of RBE group (P<0.05), indicating that M2 could impair the inhibitory effect of Lenvatinib on cholangiocarcinoma tumor growth (**Figure 8**).

### 3.7 Expression of human macrophage markers in combined M2-RBE transplanted tumors

**Figure 9:**
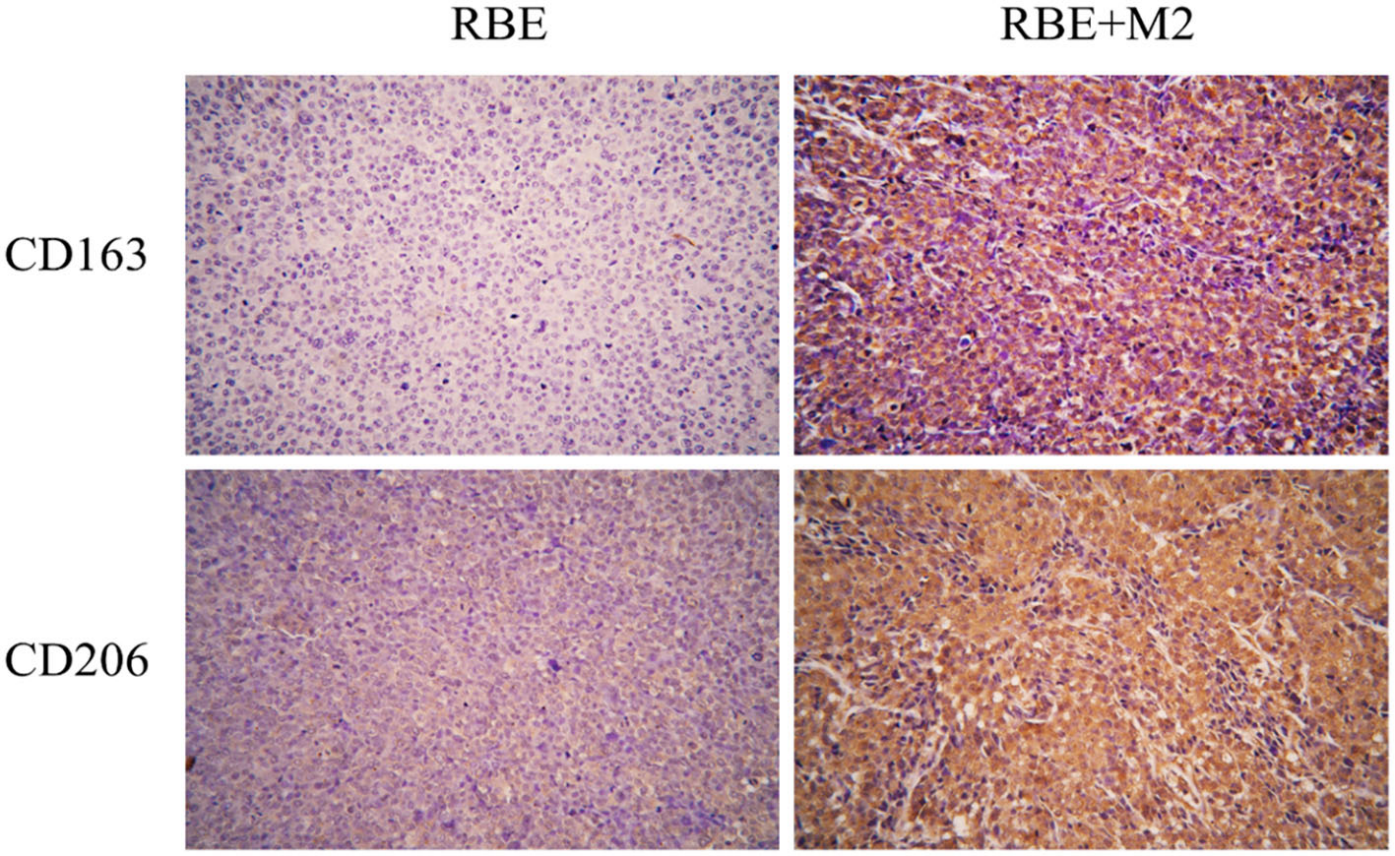
In RBE group, Subcutaneous injection 0.1ml of 3×10^7^ cells/ml RBE cell suspension, and in RBE and M2 co-culture group, Subcutaneous injection 0.1ml of 3×10^7^cells/ml RBE cell suspension and 0.1ml of 1.5×10^7^cells/ml M2 macrophage suspension. The mice in the two groups began to develop subcutaneous tumors at about 1 week, and the mice were killed by cervical dislocation 4 weeks later and remove the transplanted tumor for immunohistochemical staining. Picture for expression of CD163 and CD206 in transplanted tumor tissues in the RBE and RBE+M2 combination transplantation group.

We detected two biomarkers, CD163 and CD206, which were specifically expressed in human M2 macrophage by immunohistochemical staining. Expression of CD163 (-) and CD206 (+-) was almost absent in the subcutaneous tumor tissue of NSG mice implanted with RBE cells (**Figure 9**). The strong positive staining of CD163 (++) and CD206 (+) could be seen in the tumor tissue of RBE+M2 co-implantation group. This indicates that our induced human M2 successfully survived in NSG mice and became part of the transplanted tumor microenvironment.

### 3.8 Proliferation levels of transplanted tumor cells and expression of tumor angiogenic factors

**Figure 10:**
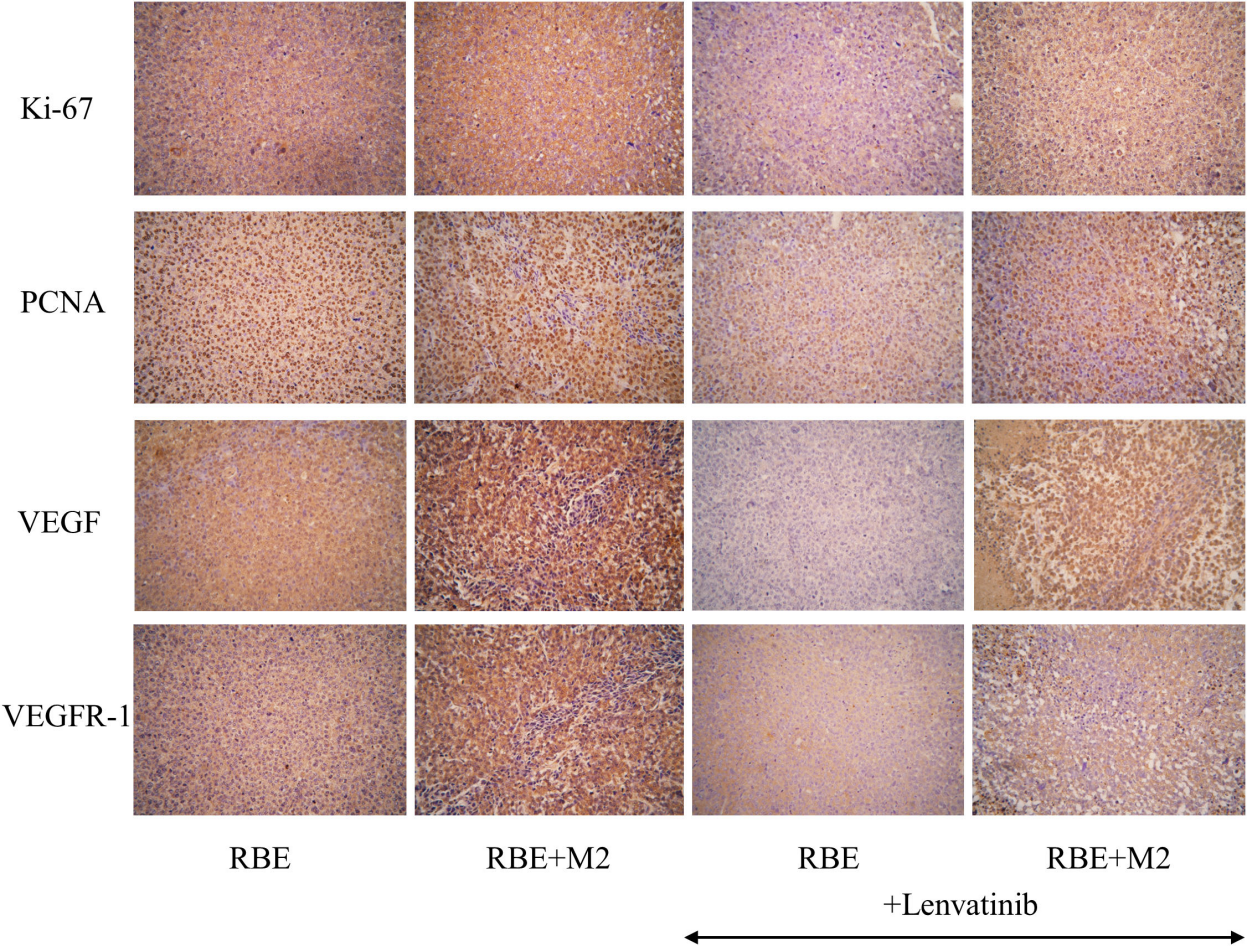
Expression of proliferative factors (Ki-67, PCNA) and angiogenic factors (VEGF, VEGFR-1) in various groups of transplant tumor cells.

As shown in **Figure 10**, the expression of Ki-67 and PCNA in the transplanted tumor tissues of the four groups were compared by immunohistochemical staining, and it was found that Ki-67 and PCNA were strongly positive in the transplanted tumors of both the RBE and RBE+M2 groups, with no significant difference between them, indicating that the proliferation of RBE cells was high and active in the tumor tissues formed by subcutaneous inoculation of RBE cells. However, their expression was reduced to varying degrees after treatment with Lenvatinib. After treatment with Lenvatinib, the positive expression rate of Ki-67 and PCNA in RBE+M2 group was significantly higher than that in RBE group, which may be related to the role of M2 in promoting tumor cell proliferation.

In addition, we detected VEGF and its receptor VEGFR-1 by immunohistochemistry to compare the tumor angiogenic ability of the four groups of transplanted tumors (**Figure 10**). VEGF and VEGFR-1 were strongly positive in RBE group and RBE+M2 group, but the staining was deeper in RBE+M2 group. After treatment with Lenvatinib, the expression of both of them decreased. After treatment, the staining intensity of VEGF and VEGFR-1 in RBE+M2 group was still significantly higher than that in RBE group, indicating that Lenvatinib could act on the VEGFR target of cholangiocarcinoma and inhibit its kinase activity, thus blocking tumor angiogenesis. On the other hand, M2 can resist the ability of Lenvatinib to inhibit angiogenesis.

### 3.9 Expression of apoptosis-related factors in transplanted tumors

**Figure 11:**
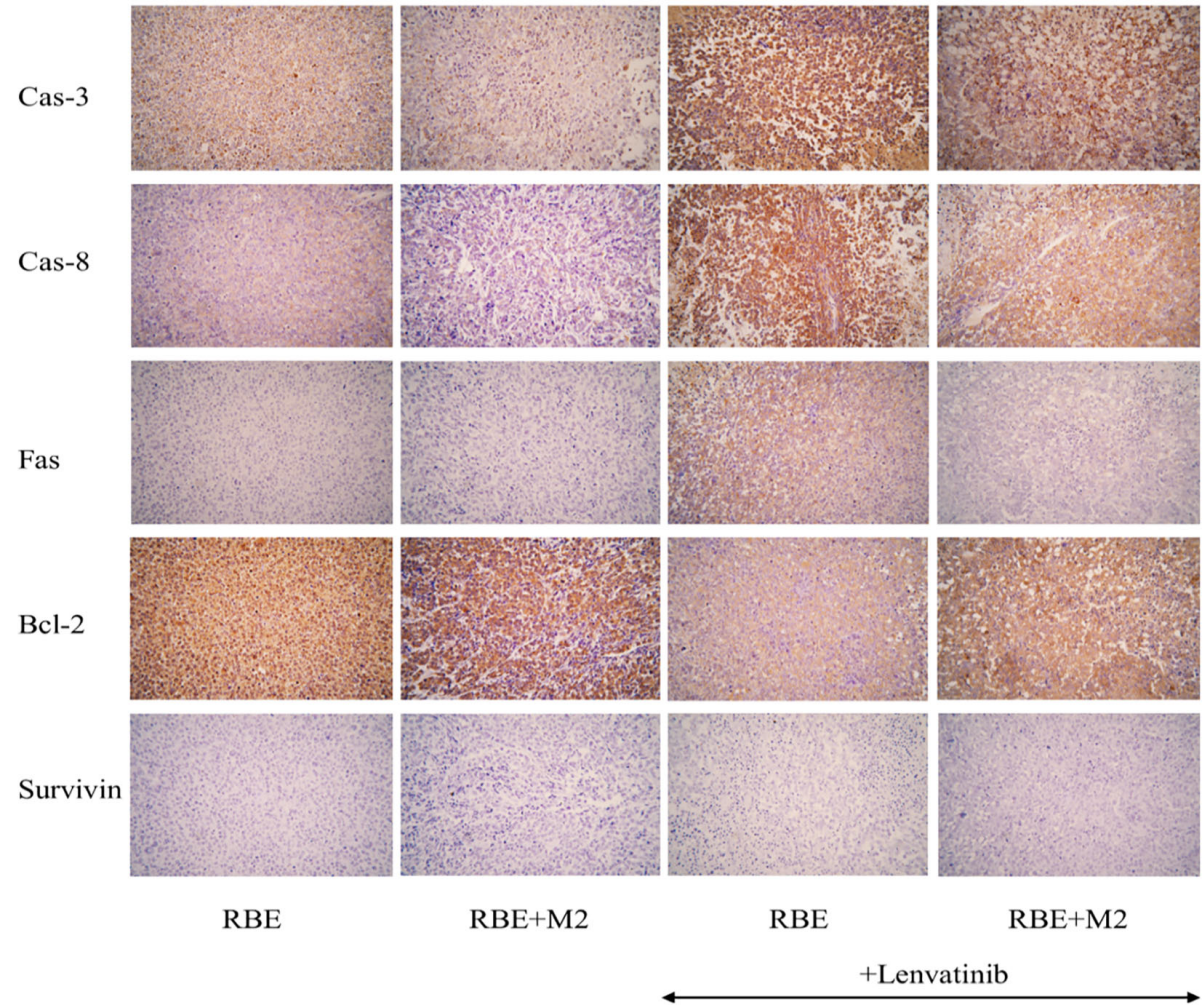
Expression of apoptosis-related factors in each group of transplant tumor cells.

We selected five apoptosis-related factors (3 pro-apoptotic factors and 2 anti-apoptotic factors) for immunohistochemical staining. The pro-apoptotic factors (Caspase-3, Caspase-8, Fas) were darker and more positive in the Lenvatinib group than in the untreated group, while the anti-apoptotic factor Bcl-2 was more positively expressed in the two untreated groups (**Figure 11**). This shows that Lenvatinib can promote the apoptosis of tumor cells. After administration, the expression of pro-apoptotic factors was less intense in the RBE+M2 group than in the RBE group, while the expression of Bcl-2 was stronger than in the RBE group, which indicated that M2 might inhibit the apoptosis-promoting effect of Lenvatinib. However, survivin, an inhibitor of apoptosis, was not significantly positive in RBE transplanted tumors.

## 4 Discussion

ICC is characterized by high malignancy, rapid growth rate, easy recurrence and metastasis, leading to a poor prognosis. For advanced unresectable ICC patients, systematic therapy based on gemcitabine or fluorouracil combined with chemotherapy, radiotherapy, and immune checkpoint inhibitor immunotherapy is often used (15, 16). Tyrosine kinase inhibitor is a kind of molecularly targeted drug commonly used in the treatment of ICC. It can exert its antitumor effect not only by inhibiting tumor neovascularization, but also by modulating the tumor immune microenvironment in combination with immune checkpoint inhibitors. As a classic tyrosine kinase inhibitor, Lenvatinib has been approved by FDA for first-line treatment of advanced hepatocellular carcinoma. Many studies have shown that it is effective for intrahepatic cholangiocarcinoma (17). Ueno M et al. demonstrated Lenvatinib as monotherapy for advanced ICC, and the ORR was 11.5% (18). The study shows that Lenvatinib monotherapy has a low response rate to ICC, and the cause of drug resistance is not clear, but it is certain that tumor immune microenvironment plays an important role.

Tumor-associated macrophages are an important part of tumor immune microenvironment (19), which are usually divided into two types, one is M1 macrophages, which are involved in inflammation and can kill cancer cells, and the other is M2 macrophage, which can promote tumor progression. Many studies have shown that, on the one hand, the abundance of macrophages in tumors is negatively correlated with the prognosis and survival of patients, and on the other hand, it is positively correlated with tumor drug resistance (20, 21). It has been previously reported that in the early stage of tumor development, macrophages in TME are mainly M1 phenotype (22). M1 macrophages are cytotoxic to cancer cells, recognizing cancer cells different from normal tissue by cell surface antigens, and then producing factors such as nitric oxide and reactive oxygen species that kill cancer cells (23). However, in the middle and later stages of malignant tumor development, in order to reduce the damage of inflammatory reaction to human normal tissue, TAM will gradually transform into M2-like phenotype (24) and promote the growth of tumor cells by secreting epidermal growth factor, fibroblast growth factor, transforming growth factor and vascular endothelial growth factor (11). M2 infiltrates and secretes a variety of cytokines in tumor tissues and plays an important role in various biological processes of tumors. Studies have shown that ICC-induced M2 can promote tumor growth and aggressiveness (25).

In addition to promoting tumor growth, M2 also has the effect of inducing tumor resistance, and some studies have confirmed that M2 can lead to drug resistance in hepatocellular carcinoma (26). Hao chen Wang et al. found that M2 can promote resistance of hepatocellular carcinoma cells against sorafenib by activating CXCR2 signaling (27). Another research has already reported that inhibiting CCR2 to block the recruitment of TAM could enhance the effect of sorafenib (28). Moreover, the hypoxia induced by target drug can elevate the level of GSF1, HIF, and CCR4 to effect M2 lead to tumor progress (29, 30). However, there are few experimental studies on TAM to ICC resistance, and in particular, there is a lack of reports of M2 effect to Lenvatinib.

In this study, we used some inducible factors to successfully induce M1 and M2 macrophages from human THP-1 monocytes. In order to study the effect of M1 and M2 macrophages on cholangiocarcinoma cells, we used Transwell chamber to culture these two kinds of macrophages without direct contact with RBE cells. THP-1 cells were implanted on the upper membrane of Transwell, which only allows the passage of soluble small molecules, and the cells cannot penetrate the membrane for migration, thus simulating the effect of macrophages on cholangiocarcinoma cells *in vivo*. According to the grouping setting, we incubated RBE cells with 3μ mol/L Lenvatinib for 24 hours after the beginning of co-culture. The protein was extracted to detect the apoptosis of RBE cells and the effect of TAM on apoptosis through paracrine factors. Through Western blot and flow cytometry analysis, we found that Lenvatinib had a significant apoptosis-promoting effect on RBE cells, while polarized M1 and M2 macrophages participated in and regulated the apoptosis-promoting effect of Lenvatinib on cholangiocarcinoma cells. M1 macrophages have cytotoxic effects and can increase the apoptosis induced by Lenvatinib. On the contrary, M2 macrophages were protective of RBE cells and inhibited their pro-apoptotic effects.

In order to further verify the effect of M2 macrophages on intrahepatic cholangiocarcinoma, we successfully constructed the cell line derived tumor xenograft model (CDX) of RBE cells in NSG mice. NSG mice is a new type of severe immunodeficiency mouse bred in recent years. It is a double knockout mouse with Rag2 and IL-2rg genes on the basis of NOD/scid mice. Its own T hand B cells and NK cells are absent, and the function of dendritic cells and macrophages is also defective (31). Significantly higher survival of human immune cells and tumor cells transplanted in NSG mice could be required for the reconstruction of a human immune system model. We observed that the transplanted tumor grew more rapidly in mice transplanted with RBE cells and polarized M2 macrophages. Immunohistochemical staining showed strong positivity for CD163 and CD206, specific markers of human M2 macrophages. It suggested that M2 macrophages successfully survived in NSG mice and were widely present in the microenvironment of transplanted tumors.

By comparing the volume of transplanted tumors in each group, we found that Lenvatinib could significantly inhibit the growth of intrahepatic cholangiocarcinoma xenografts, while the addition of M2 macrophages could weaken this antitumor effect. Then we observed that the positive rates of Ki-67 and PCNA in RBE+M2 group were significantly higher than those in RBE group by immunohistochemical staining, which may be related to the role of M2 macrophages in promoting tumor cell proliferation. In addition, we detected the expression of five known apoptosis-related factors in transplanted tumor and found that the expression intensity of pro-apoptotic factors (Caspa-se-3, Caspase-8, Fas) in RBE+M2 group was lower than that in RBE group, while the expression of anti-apoptotic factor Bcl-2 was stronger than that in RBE group. This indicated that M2 macrophages could inhibit the pro-apoptotic effect of Lenvatinib, and the *in vivo* results were consistent with those of cell experiments. Previous studies have reported that TAM may attenuate the apoptosis-inducing effect of sorafenib in hepatoma cells by increasing autophagy (32), which is similar to our findings. In the future, we can further explore the mechanism of M2 macrophages inhibiting ICC apoptosis by designing gene knockdown mice, so as to provide new targets and clinical strategies for the treatment of ICC.

In the previous study, through the establishment of a PDX library of primary liver cancer, we found that the tumor formation rate of liver cancer tissue transplanted into nude mice was low, especially since the tumor formation of hepatocellular carcinoma was so unstable, making it difficult to achieve clinical translation of PDX as a drug screening model. According to the results of this study, we can hypothesize that if we construct the PDX model using NSG mice and add human M2 macrophages during the modeling process, it will inevitably increase the tumor formation rate, decrease the tumor formation time, and save the cost of sub generational modeling. However, drug screening experiments are still needed to verify the consistency of this model with the traditional PDX model.

## Data availability statement

The original contributions presented in the study are included in the article/supplementary materials, further inquiries can be directed to the corresponding authors.

## Ethics statement

Ethical approval was not required for the studies on humans in accordance with the local legislation and institutional requirements because only commercially available established cell lines were used. The animal study was approved by Tianjin Tiancheng New Drug Evaluation Co., Ltd. Laboratory Animal Use Management Committee. The study was conducted in accordance with the local legislation and institutional requirements.

## Author contributions

LY, YZ, and JZ conceived the study. LY and PH drafted the manuscript. LY, TC, YM, and TZ performed the literature search and collected the data. ZC, HC, and YC helped with the final revision of this manuscript. All authors reviewed and approved the final manuscript. All authors contributed to the article and approved the submitted version.
